# Genetic structure and demographic inference of the regular sea urchin *Sterechinus neumayeri* (Meissner, 1900) in the Southern Ocean: The role of the last glaciation

**DOI:** 10.1371/journal.pone.0197611

**Published:** 2018-06-06

**Authors:** Angie Díaz, Karin Gérard, Claudio González-Wevar, Claudia Maturana, Jean-Pierre Féral, Bruno David, Thomas Saucède, Elie Poulin

**Affiliations:** 1 Departamento de Zoología, Universida d de Concepción, Barrio Universitario s/n, Concepción, Chile; 2 Facultad de Ciencias, Universidad de Magallanes, Bulnes, Punta Arenas, Chile; 3 Laboratorio de Ecología Molecular Antártica y Subantártica, Universidad de Magallanes, Punta Arenas, Chile; 4 Instituto de Ciencias Marinas y Limnológicas, Facultad de Ciencias, Universidad Austral de Chile, Valdivia, Chile; 5 Instituto de Ecología y Biodiversidad (IEB), Departamento de Ciencias Ecológicas, Facultad de Ciencias, Universidad de Chile, Las Palmeras, Ñuñoa, Santiago, Chile; 6 UMR 7263—IMBE, Station Marine d’Endoume, Institut Méditerranéen de Biodiversité et d’Ecologie Marine et continentale, Chemin de la Batterie des Lions,Marseille, France; 7 Biogéosciences, UMR CNRS 6282, Université de Bourgogne, boulevard Gabriel, Dijon, France; 8 Museum National d’Histoire Naturelle, Paris, France; National Cheng Kung University, TAIWAN

## Abstract

One of the most relevant characteristics of the extant Southern Ocean fauna is its resiliency to survive glacial processes of the Quaternary. These climatic events produced catastrophic habitat reductions and forced some marine benthic species to move, adapt or go extinct. The marine benthic species inhabiting the Antarctic upper continental shelf faced the Quaternary glaciations with different strategies that drastically modified population sizes and thus affected the amount and distribution of intraspecific genetic variation. Here we present new genetic information for the most conspicuous regular sea urchin of the Antarctic continental shelf, *Sterechinus neumayeri*. We studied the patterns of genetic diversity and structure in this broadcast-spawner across three Antarctic regions: Antarctic Peninsula, the Weddell Sea and Adélie Land in East Antarctica. Genetic analyses based on mitochondrial and nuclear markers suggested that *S*. *neumayeri* is a single genetic unit around the Antarctic continent. The species is characterized by low levels of genetic diversity and exhibits a typical star-like haplotype genealogy that supports the hypothesis of a single *in situ* refugium. Based on two mutation rates standardized for this genus, the Bayesian Skyline plot analyses detected a rapid demographic expansion after the Last Glacial Maximum. We propose a scenario of rapid postglacial expansion and recolonization of Antarctic shallow areas from a less ice-impacted refugium where the species survived the LGM. Considering the patterns of genetic diversity and structure recorded in the species, this refugium was probably located in East Antarctica.

## Introduction

The marine benthic fauna of the Southern Ocean is unique and considered to be the most isolated on the planet [1,2,3,4,5]. Indeed, most invertebrate and fish species from the Southern Ocean are highly endemic (75–90%) [6,7,8,9]. The Southern Ocean comprises the most southerly basins of the Atlantic, Indian and Pacific Oceans, across which flows the Antarctic Circumpolar Current (ACC). The ACC has a series of eastward flowing jets that extend to 3000–3500 m depth, and is delimited northward and southward by two convergence zones, the Subantarctic Front (SAF) and the Antarctic Polar Front (APF). The ACC and the APF represent an important boundary in terms of high current speeds and strong horizontal gradients in density, temperature, salinity and air-sea fluxes [10]; they isolate the Antarctic continent and its associated archipelagos and islands from the other continental landmasses [11].

The gradual onset of colder conditions with subsequent large-scale glaciation in Antarctica since the early Oligocene has been pivotal to shape the evolution of the Antarctic biota [12,13,14,15,5,16]. The deepening seaway through the Drake Passage, resulting from the separation of South America and Antarctica (~ 30 Mya), completed the isolation of the Antarctic continent and initiated the ACC [1,17,18]. A marked decline in temperature during the mid-Miocene (c. 14 Mya), induced the re-establishment of a permanent continental ice sheet in East (10 Ma) and West (5 Ma) Antarctica [19]. Antarctic continental glacial ice sheet expansions have been associated with one of the most important extinctions on the continent and therefore of the evolution of the Antarctic marine life [13,20,21]. In fact, several groups of marine organisms that are highly abundant and diverse in adjacent areas have become extinct in the Southern Ocean (e.g. brachyura, chondrichtyes [15]) while other groups have become very abundant (e.g. Brachiopoda) and/or more diverse (e.g. Pycnogonida) than elsewhere [11]. Extreme climate conditions, the geographic isolation of the continent, major oceanic currents and the bathymetry all act as strong barriers surrounding the Antarctic Continent, thus explaining the high levels of endemism recorded in the Antarctic marine benthic biota.

At least 38 glaciation cycles have been identified over the last 5 million years by sediment core data [22,23]. The advance of grounded ice sheets across the Antarctic shelf and the associated mass-wasting processes on the continental slope caused major disturbances of an order of magnitude greater than those recorded currently by iceberg plowing [24]. However, most of the evidence concerning Quaternary glacial effects comes from the Last Glacial Maximum (LGM), with which the glaciological models associated a massive advance of Antarctic ice sheets, a fall in global sea levels and a grounding of ice sheets out across much of the continental shelf of the Southern Ocean and up to 100 km on the continental slope [25,26]. At higher latitudes, ice sheet advances and lower global temperatures during glacial maxima removed shelf communities and reduced the spatial distribution of species thus affecting population sizes [27] and patterns of intraspecific genetic variation [28,29,30,31,32,33,17]. Moreover, some studies have shown that the glacial and interglacial cycles are also responsible for the diversification of several Antarctic groups including nemerteans, mollusks, echinoderms and crustaceans [34,35,36,37,38]. The “species diversity pump” hypothesis, proposed by Clarke and Crame [39,40], suggests that successive ice advances and retreats over the shelf had a significant impact on the marine diversity in polar and subpolar areas, since they could favor speciation by the repeated isolation and reconnection between faunas. This process would have been particularly effective for species with limited dispersal capabilities such as those with non-pelagic development [41,35].

However, the response of species to climate change does not necessarily depend on life-history traits, but also on the availability of suitable habitats, that may not allow the species to survive. For marine species living on the Antarctic continental shelf, glacial periods would have corresponded to maximum contraction in geographical range [42,43]. The glacial periods have attracted the attention of several Southern Ocean evolutionary biologists, in particular regarding the strategies developed by the marine benthic fauna to deal with ice sheet expansion and contractions [44,45,13,46]. Three main hypotheses have emerged to explain how species survived through glacial maxima: (i) *In situ* persistence in Antarctic glacial refugia; (ii) Survival in peri-Antarctic islands or in sub-Antarctic areas; (iii) Retreat to the shelf slope and deep-sea habitats. The *in situ* persistence scenario suggests the presence of one or several isolated refugia on the shelf associated with strong population bottlenecks. Under this scenario, a species should be characterized by low levels of genetic diversity and by a star-like haplotype network with short genealogies in the case of a single refugium [24,47]. More complex networks and higher levels of genetic diversity are expected in the case of multiple refugia [17]. A scenario of survival in peri-Antarctic islands or in sub-Antarctic areas is comparable to the expansion-contraction (EC) model of Pleistocene biogeography proposed by Provan & Bennet [48]. It assumes that the distributions of taxa with narrow bathymetric ranges are contracted toward lower latitudes glacial refugia where the shallow habitats would have been less impacted during periods of cooling. In this scenario, the current Antarctic populations experienced demographic growth after post-glacial recolonization and have low genetic diversity as well a signal of demographic expansion [32,46,49]. In contrast, lower latitude populations should exhibit higher levels of genetic diversity. This hypothesis provides a satisfactory explanation for two species of benthic fish of the continental shelf, *Trematomus bernacchii* and *Trematomus pennelli* [50], and there is good evidence that South Georgia acted as a refugium for the Antarctic limpet *Nacella concinna* [33] and for the sea spider *Colossendeis megalonyx* [51]. The retreat onto the shelf slope and deep-sea habitats scenario assumes that Antarctic shelf species survived in the deeper part of their bathymetric distributions in less ice-impacted areas [45,52], and then recolonized the shelf during the deglaciation process. The switch to deeper areas may have occurred at a very large geographic scale, maintaining large population size and high genetic diversity, as well as highly divergent lineages as shown for the nudibranch *Doris kerguelensis* [36] and the pycnogonid *Nymphon australe* [30,53]. In some cases, also propose several glacial refuges have been proposed for circum-polar deep-sea species such as the crinoid *Promachocrinus kerguelensis* [54].

One of the most conspicuous and endemic inhabitants of the Antarctic continental shelf is the regular sea urchin species *Sterechinus neumayeri* (Meissner, 1900). This species is found from the shallow subtidal zone down to 500 m depth, but is mainly distributed in the inner and mid continental shelf [55,56]. It has a circum-Antarctic distribution, although it has been reported in sectors bordering the Antarctic convergence such as the South Georgia [57]. However, South Georgia *S*. *neumayeri* individuals were identified as *S*. *agassizii*, a congeneric species with a broad distribution in the Antarctic and Subantarctic (Chester Sands, personal comment). *Sterechinus neumayeri* is a broadcast-spawner with free-living larvae that settles in the water column after 4 months [58]. Due to its abundance and its circum-Antarctic distribution, *S*. *neumayeri* represents a suitable model for studies in reproductive biology [59], embryology [58,60,61], ecology [62,63,64,65,66], physiology [67,68] and toxicology [69]. However, little is know about the evolutionary history of the species and its ecological success that allows its wide distribution and abundance across the Antarctic continent. So far, the endemic distribution of *S*. *neumayeri* on the Antarctic continental shelf has been associated with depth, sea ice coverage and sea surface temperature [55], but the glacial–interglacial periods and particularly the LGM should be the key to understand its distribution and evolutionary history.

Molecular approaches are helping us to understand patterns of genetic diversity and structure better and to infer historical and contemporary demographic processes. Díaz et al. [31] described the absence of phylogeographic structure in samples of *S*. *neumayeri* from the Western Antarctic Peninsula (WAP) and East Antarctica (Adélie Land) that could reflect the existence of past or present connectivity sufficient to impede any divergence processes around the continent. Considering that the extension of ice sheets during the LGM may have dramatically affected *S*. *neumayeri* populations in shallow waters, the lack of phylogeographic structure could rather be a consequence of a postglacial demographic expansion scenario following a bottleneck or even to a founder effect related to postglacial recolonization. In this study, we analyzed the effect of the recent climatic history of the Southern Ocean on the evolutionary history of *S*. *neumayeri* through genetic structure and demographic inference analyses, using both mtDNA and microsatellites markers.

## Materials and methods

### Ethics statement

This study was conducted using *Sterechinus neumayeri* as study model, a common sea urchin species from the continental shelf of the Antarctic Continent. The species is considered as an Antarctic marine living resource; therefore it is protected by the Commission for the Conservation of Antarctic Marine Living Resources CCAMLR. Permission to collect specimens was issued by the Chilean Antarctic Institute (INACH). Instituto de Ecología y Biodiversidad (IEB/15-2015) ethics committee, which approved sampling protocols and experiments. We complied with local legislation and the Convention on Biological Diversity.

### Samples and DNA extraction

**Fig 1:**
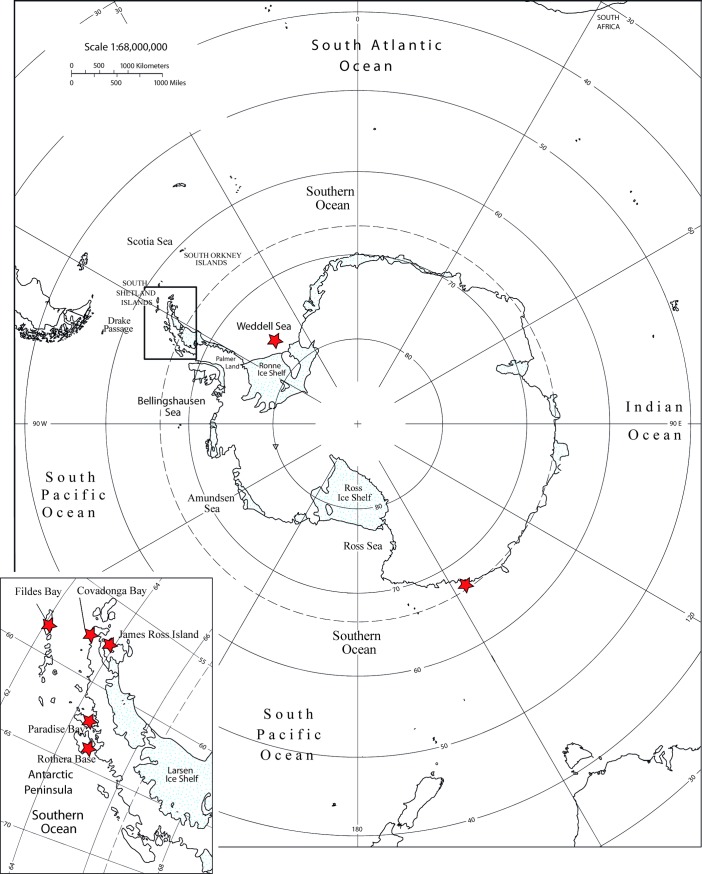
Map of localities. Localities analyzed of the species *Sterechinus neumayeri* in Antarctica.

Individuals were collected between 2004 and 2012 in shallow waters at four localities across two major regions including West Antarctica: a) Antarctic Peninsula (AP): James Ross Island, East AP (JRI 64°18´53"S—57°07´53" W: n = 8); Paradise Bay, West AP (PB 64°51´05"S—62°54´36" W: n = 10); Rothera Station, Adelaide Island, West AP (RO 67°42´55”S—68°04´30” W: n = 19); and b) the Weddell Sea (WS 71°56´02"S—47°59´53" W: n = 6; Fig 1). Whole specimens were fixed in ethanol (95%), and DNA was extracted from the gonads using a salting-out method described by Aljanabi & Martinez [70]. We also included in the analyses samples/sequences from Adélie Land (AL 65°59´11"S—139°36´41" E: n = 15), East Antarctica described by Díaz et al. [31], and samples/sequences of three localities from AP described by Díaz et al. [31], González-Wevar et al. [71] and Poulin et al. [72]: Fildes Bay, King George Island, West AP (FB 62°12’S; 58°56’W: n = 66), Covadonga Bay, West AP (CB 63°22’S; 58°09’W: n = 44) and James Ross Island, East AP (JRI 64°18´53"S—57°07´53" W: n = 6; Fig 1) (ACCN: KJ571184–KJ571197). Unpublished COI sequences of *S*. *neumayeri* were deposited in GenBank under the accession numbers MG783406-MG783567.

### Mitochondrial sequencing and analyses

A partial fragment of the mitochondrial gene cytochrome c oxidase subunit I (COI) was amplified with specific primers following PCR conditions described by Díaz et al. [31]. PCR amplicons were purified using QIAquick Gel Extraction Kit (QIAGEN) and sequenced in both directions using an Automatic Sequencer 3730 x 1 Automatic Sequencer at Macrogen Inc. (Seoul, Korea). Chromatograms were edited using Proseq version 3.5 [73] and the final alignment of 931 base pairs was made with ClustalW [74]. Sequences were translated to amino acids to check for the presence of pseudogenes and/or sequencing errors with MEGA 6.0 [75]. We performed a DNA saturation analysis comparing the sequences with an expected rate using the DAMBE 4.5.27 program [76].

#### Genetic diversity and population structure

Levels of genetic polymorphism were determined using standard diversity indices: number of haplotypes (k), number of segregating sites (S), haplotype diversity (H), average number of pairwise differences (Π) and nucleotide diversity (ᴨ) for each locality, for each region and for the whole COI sequence set using DnaSP, version 5.00.07 [77]. We performed neutrality tests (Tajima’s D and Fu’s F_S_) for each region and for the whole dataset to measure whether data deviate from expectations under a mutation-drift model with DnaSP version 5.00.07 [77]. To have comparable observations among localities with different numbers of individuals, we performed haplotype accumulation curves per locality based on species richness; these estimators were calculated from rarefaction curves using 999 randomizations and sampling without replacement using iNEXT [78]. Extrapolations were made based on 100 individuals and we compared richness among 41 samples (RAR41). Species richness was considered significantly different among sites when 95% confidence intervals did not overlap between them for 41 samples. To infer the spatial genetic structure of *S*. *neumayeri*, a clustering method was used to estimate the number and the composition of panmictic groups, as well as the spatial boundaries between them using a Bayesian model computed with the GENELAND package, version 4.0.0 [79] in the R environment (R, version 2.4.1; [80]). This software implements a Markov Chain Monte Carlo (MCMC) procedure to determine the best clustering of samples with regard to genetic and geographical information. Geographical information is taken into account at the Bayesian prior level, so that clusters corresponding to spatially structured groups are considered to be more likely than clusters that are randomly distributed in space. Five x 10^6^ MCMC iterations were sampled each 1000 steps with a 500 step burn-in period, and a maximum number of clusters K = 10 were run to estimate the model parameters and posterior probabilities of group membership.

#### Demographic inference

The genealogical relationships among COI haplotypes for each region and for the whole dataset of *S*. *neumayeri* were characterized using median-joining networks computed with the program Network 5.0.0.1. [81]. Past population demographic growth was evaluated by comparing the distribution of pairwise differences between haplotypes (mismatch distribution) to the expected distribution under the sudden expansion growth model of Rogers & Harpending [82]. In addition, we estimated past population dynamics through time using the Bayesian skyline plot method implemented in BEAST, 1.7 [83]. We conducted two independent Bayesian MCMC runs using the generalized time-reversible (GTR) with a gamma distribution (G) and a proportion of invariable sites (I); the GTR+G+I model (ref GTR: [84]), previously estimated with JModelTest 3.06 [85], for two mutation rates calibrated for COI sequences of sea urchins of the Infraorder Echinidea by Lee et al. [86]: 0.51 and 0.72% Myr^-1^. Substitution rates were modified to a tenfold evolutionary rate (10% per million years: 5.1 and 7.2%), considering the correction for time dependence of molecular rates at the population level proposed by Ho et al. [87]. It is indeed necessary to distinguish between the interspecific phylogeny-based substitution rate and population mutation rate based on intraspecific gene or genealogy-based analyses [88,89]. Genealogy-based mutation rates have been estimated through pedigree or ancient DNA studies analyses, and are generally much greater than fossil-calibrated substitution rates [87,90,91]. Even though the time dependence of molecular rates correction is still under controversy [92,93], it has been broadly implemented in population-based studies of Antarctic organisms such as algae [94], Antarctic limpets [32] and Gentoo penguins [95]. The two independent MCMC calculations were run for 3,5 x10^8^ generations (sampled every 1000 iterations), discarding the first 10% of the trees as burn-in. The convergence of runs was confirmed with Tracer v1.0.1 [83], ensuring a minimum of 1000 effective samplings for each statistic (ESSs). The median and corresponding credibility intervals of the Bayesian skyline plot were depicted with Tracer.

### Microsatellite analyses

Microsatellite loci were developed by ATG GENETIC INC. using genomic enrichment and plasmid cloning methodologies [96]. Briefly, genomic DNA was digested using restriction endonucleases (Hae III or Rsa I with PshA I) and simultaneous ligation to the synthetic adaptor M28/M29P with T4 DNA ligase. Microsatellite DNAs was then enriched through two rounds of hybridization with biotin-labelled synthetic oligos and captured with streptavidin magnetic beads using two sets of microsatellite classes (bTG12 and bGA12, bGATA5 and bCATA5). Doubly enriched microsatellite DNA fragments were digested with EcoR1 (G/AATTC) and ligated into the plasmid vectors (pGEM3z+, Promega). Positive microsatellite clones were identified by dot blot hybridization with appropriate mixes of biotin labelled SSR oligonucleotides. Microsatellites were then sequenced in an ABI 3730 sequencer (Applied Biosystems), using M13 universal forward and reverse primers, treated with Exonuclease I and shrimp alkaline phosphatase. Eighteen successfully sequenced clones contained repeat motifs (di, tri and tetranucleotide). Primers were designed for all the positive clones either manually or using Primer 3 [97]. Eight microsatellites were scored as ‘‘useful” based on good amplification of polymorphic sized bands from single copy genomic targets, but only five of them gave polymorphic PCR results and were considered robust and predictable enough for further analyses (Table 1).

**Table 1:** Description of the microsatellite loci used in the study.

Locus name	Primer sequence (5’– 3’)	Repeat Motif (SSR)	Size range (bp)	T_a_ (°C)
F32St	F: TGAAATTCGTCGTCAACACA	(CTT)8	163–181	Td^a^
	R: CGTTACTACCGGGGACACTG			
P1St	F: TTAAAGACATTCCCTGTTTCATCA	(TC)8	144–154	58
	R: ACACACACACACTTTCCCACTC			
P2St	F: CACACCCCACACACTCTCTCT	(AG)12	120–158	Td^a^
	R: TGTGGAAAATGTGCGTGAGT			
N4St	F: ACTCACGCACATTTTCCACA	(GA)10	222–228	58
	R: GAGGTCGGGAGAGATTCTGA			
V6St	F: GTGCATGTATGAGCTGGCTC	(ATT)10	250–295	58
	R: CCTGTTGCGCAAACAGCAAG			

Locus name; Primer sequence; SSR (Short Sequence Repeat) motif; Size range in number of base pairs (bp); Annealing temperature (T_a_).

^a^ Td: Touchdown PCR, see explanation in the text.

Polymerase chain reaction (PCR) amplification mixtures (15 μl) contained 10 ng template DNA, 5 pmol each primer, 250 μM dNTPs, 1.5 μL 10 X PCR buffer and 0.5 U Taq DNA polymerase (Invitrogen). Cycling conditions consisted of an initial denaturing step of 5 min at 94°C, followed by 35 cycles of 1 min at 94 °C, 30 s at the specific annealing temperature (Table 1), 1 min at 72 °C and a final elongation step at 72 °C for 10 min. For loci F32St and P2St we used a touchdown program (Td) in which the annealing temperature is gradually reduced. This consisted of an initial denaturing step of 5 min at 94 °C, followed by 25 cycles of 30 s at 94 °C, 45 s at 60 °C with decrease of 0.5° every two cycles ending with 30 s at 72 °C, followed by 10 cycles of 30 s at 94 °C, 45 s at 50 °C and 30 s at 72 °C and a final elongation step at 72°C for 10 min. PCR products were analyzed in an ABI-PRISM 3730xl Analyzer (Roy J. Carver Biotechnology Center) using the LIZ 500 Size Standard (Applied Biosystems). Alleles were identified using PEAK SCANNER Software v.1 (Applied Biosystems).

Because it was not possible to amplify the Weddell Sea samples at any loci, we evaluated the genetic differentiation between Antarctic Peninsula localities (West Antarctica) and Adélie Land (East Antarctica) for a total of 73 individuals. This reduced number of individuals was due to difficulties in the amplification of each locus (probably associated with the presence of degraded DNA).

#### Genetic diversity and Hardy–Weinberg equilibrium

Genetic diversity analyses were conducted on 73 individuals. The presence of null alleles and scoring errors was checked with MICROCHECKER v.2.2.3 [98]. All non-amplifying genotypes were checked by re-amplification at least twice [99]. All loci were tested for linkage disequilibrium and for deviation from Hardy–Weinberg equilibrium (HWE) by means of a permutation test implemented in the software GENETIX version 4.05.2. [100]. Observed (Ho) and expected (He) heterozygosity and allele number (Na) were also calculated with GENETIX. Robust Multilocus Estimation of Selfing (RMES) software [101] was used to reveal biparental inbreeding in the regions. The inbreeding rate (s) was deduced from ĝ_2_, an estimator of the two-locus heterozygosity disequilibrium over all pairs of loci, under the assumption of inbreeding and linkage equilibrium. P-values for the null hypothesis s = ĝ_2_ = 0 were obtained by resampling single-locus heterozygosity among individuals within the regions 1000 times (α = 0.05).

#### Genetic structure analyses

Two independent analyses were performed for the structure analyses between the two major biogeographic areas of the Antarctic continent. The fixation index (F_ST_) was estimated in GENETIX for each locus separately and overall, and tested for significant differentiation from zero by a permutation test also implemented in GENETIX. These estimates and tests were conducted 1) considering the whole dataset (73 individuals) and 2) after removing individuals with missing data for two loci (leaving 39 individuals). STRUCTURE v2.3.3 [102], was used to identify the number of populations or genetic clusters in *S*. *neumayeri* around Antarctica. Two different runs were done with K from 1 to 5; a first run with the whole dataset and a second run with 65 individuals (individuals with 2 missing loci data were excluded), in order to reduce the possible error that can be inferred from the amount of missing data. Eleven independent replicates with one million Markov chain Monte Carlo (MCMC) analyses were performed for each run with a 100,000 burn-in period under the “admixture model”.

## Results

### Mitochondrial evidence

#### Genetic diversity and population structure

We included in the analyses a total of 174 individuals of *Sterechinus neumayeri* from seven localities across three regions around the Antarctic continent. A fragment of 931 base pairs coding for 310 amino acids of the mtDNA COI gene was amplified, corresponding to nucleotides 79 to 1008 of the gene (Genbank accession AY275548, total size 1077 bp). No insertion/deletion or stop codons were detected in the sequence set. Sequences were not saturated at any position and two amino acid changes were recorded (translated using the invertebrate mitochondrial table; [103]). At position 68, a transversion (C–A) generated an amino acid change from isoleucine to methonine (both have a hydrophobic side chain), and at position 135, a transversion in the second position (C-A) generated an amino acid change from serine to tyrosine (both amino acids have polar side chains). As previously recorded in *Sterechinus* genus [31], sequences of *S*. *neumayeri* were A-T rich (57.6%) compared to the mean G-C content (42.4%). *Sterechinus neumayeri* exhibited low levels of genetic diversity across the Antarctic continent, even though at first glance Fildes Bay in Antarctic Peninsula exhibited higher levels of diversity measured as the number of polymorphic sites (S) and the number of haplotypes (k). Rarefaction analysis showed similar values of haplotype number among localities of Antarctic Peninsula except in James Ross Island and Paradise Bay (RAR41 = 2 haplotypes ± 0.9, for both localities) and, Adélie Land showed a higher haplotype number than the rest localities (and regions). The Weddell Sea population showed only two polymorphic sites and three haplotypes while Adélie Land in east Antarctica exhibited higher levels of genetic diversity. Haplotype diversity (H), average number of nucleotide differences (Π) and mean nucleotide diversity (ᴨ) were low in the three regions but the lowest values were detected in the Antarctic Peninsula. Despite being the region with the lowest number of samples analyzed, the Weddell Sea showed the highest levels of haplotype diversity. Similarly, Adélie Land showed the highest levels of average number of nucleotide differences, as well as nucleotide diversity (Table 2).

**Table 2:** Genetic diversity indices and neutrality tests in *Sterechinus neumayeri*.

Locality	N	K	RAR_41_		S	H	Π	ᴨ	Tajima’s D	Fu’s F_s_
J. Ross Island	14	2		2.0±0.9	1	0.143±0.119	0.143	0.00015	-	-
Covadonga Bay	44	5		4.7±2.2	4	0.175±0.077	0.182	0.00020	-	-
Paradise Bay	10	2		2.0±0.9	1	0.200±0.154	0.200	0.00021	-	-
Rothera Base	19	4		5.3±3.4	3	0.380±0.134	0.409	0.00044	-	-
Fildes Bay	66	9		5.9±2.8	9	0.229±0.069	0.273	0.00029	-	-
**R**_1_ Antarctic Peninsula	153	18		-	18	0.222±0.046	0.248	0.00027	-2.499**	-7.177*
**R**_2_ Weddell Sea	6	3		3.8±2.1	2	0.600±0.215	0.667	0.00072	-1.132	-1.155
**R**_3_ Adélie Land	15	5		8.9±5.0	8	0.476±0.155	1.067	0.00115	-2.086*	-2.769*
*S*. *neumayeri* TOTAL	174	23		-	24	0.257 ±0.045	0.333	0.00036	-2.567**	-6.536*

n: number of sampled individuals; k: number of haplotypes detected; RAR_41_: number of haplotypes by rarefaction; S: polymorphic sites; H: haplotype diversity; Π: average number of nucleotide differences; ᴨ: mean nucleotide diversity. Neutrality test levels of significance

** P < 0.01

* P < 0.05.

The model based on the Bayesian clustering algorithm implemented in Geneland detected a single cluster for the data set (K = 1), with high values of posterior probability of cluster membership (c.a. P = 0.7), suggesting that there is no genetic structure for the species around Antarctica.

#### Demographic inference

**Fig 2:**
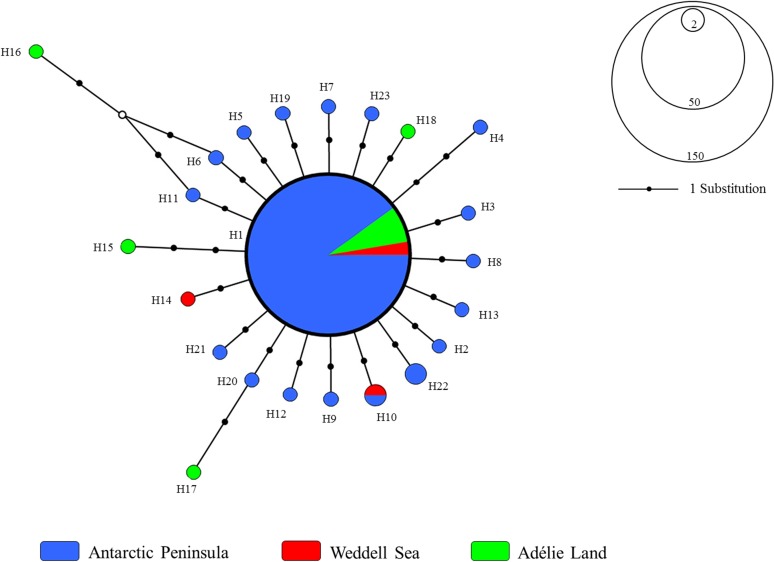
Network. General median joining haplotype network including 174 *Sterechinus neumayeri* mtDNA COI sequences. Each haplotype is represented by a colored circle indicating the main area where it was collected and the size of the circle is proportional to its frequency in the whole dataset. ° = median vector (theoretical haplotype that has not been collected but should exist).

Considering the whole COI data set as single genetic unit (Geneland k = 1), the median-joining haplotype network of *S*. *neumayeri* included 23 different haplotypes and showed a typical star-like topology with a very short genealogy: a central haplotype occurring in all regions in high frequency (H1 = 85.1%), from which many low frequency haplotypes radiate separated by one (for the majority) to 2 substitutions. A total of 21 singletons were recorded in the species (Fig 2). As expected for star-like topologies, general Tajima’s D and Fu’s F_S_ indices were negative and significant, showing significant deviation from mutation-drift equilibrium for the whole data set.

**Fig 3:**
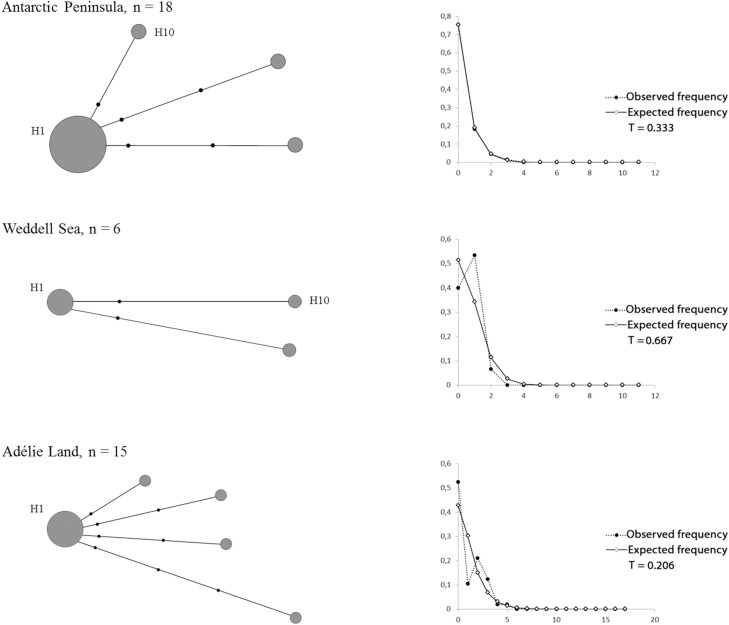
Networks for each area. Median-joining haplotype networks and the distribution of mean number of pairwise differences (mismatch) for each area included in the analyses. *To compare among areas, 18 individuals were randomly selected from the Antarctic Peninsula.

The haplotype network constructed for each region separately showed similar star-like topologies and very short genealogies. All the regions shared a central dominant haplotype of high frequency broadly distributed at all sampling sites (H1: 86.9% AP, 66.6% WS and 73.3% AL) and a second haplotype of low frequency recorded at in the Antarctic Peninsula and the Weddell Sea (H10, Fig 3).The Adélie Land region showed a greater number of haplotypes than the other two regions and the presence of several private haplotypes (4 vs 2 and 1 for AP and WS, respectively). This locality also showed an expanded genealogy by having a greater number of nucleotide substitutions between haplotypes (Π values, Table 2). Tajima’s D and Fu’s F_S_ neutrality tests were negative for the three regions as expected for star-like topologies, but were significant only for AP and AL (Table 2). Similarly, as expected for star-like topologies, the mismatch distributions for the regions followed an L-shaped positively skewed and unimodal distribution with minimal differences among them (Fig 3).

**Fig 4:**
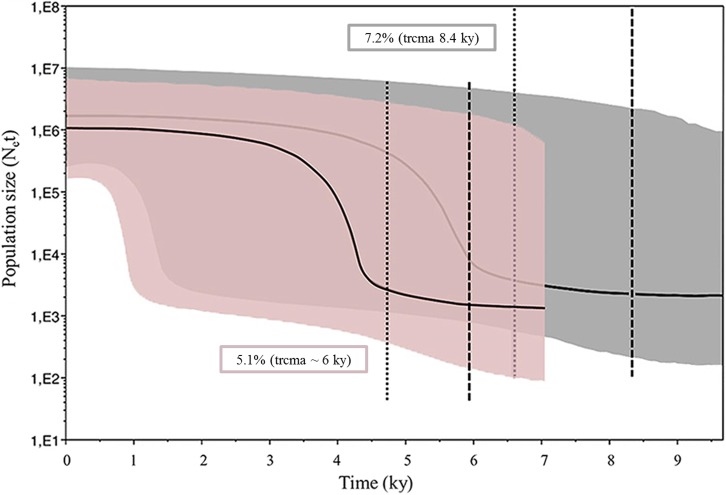
Historical demographic trends. Historical demographic trends of effective population size (Ne) constructed using a Bayesian skyline plot approach based on COI haplotypes of *S*. *neumayeri*, with two mutation rates (5.1% and 7.2%). The y-axis is the product of effective population size (Ne) and generation length in a log scale while the x-axis is the time in 10^3^ before present. The median estimate (solid black line) and 95% highest probability density (HPD) limits (gray/pink area) are shown. The thick dashed line represents the time of the most recent ancestor (trcma) and the thin dashed line represents time for the expansion in the species.

Bayesian Skyline plot analysis supports a recent population expansion of *S*. *neumayeri* across the Antarctic continent. The time for the most recent common ancestor (trmca) appears between 6–8.4 ky while the onset of the population expansions occurred between 4.7–6.6 ky (Fig 4).

### Microsatellites

#### Genetic diversity and Hardy–Weinberg equilibrium

A total of 32 alleles were detected across loci in the 73 genotypified individuals. All the analyzed loci were polymorphic in the species. Exact tests for linkage disequilibrium yielded no significant values (P>0.05). Overall heterozygote deficiency (F_IS_ = 0.128) was significant (p < 0.001), revealing a strong deviation from HWE. Significant departure from HWE was detected in three of the five loci along the Antarctic Peninsula (P1St–P2St–N4St) while two loci were significant for the east Antarctica AL (P1St–P2St), revealing heterozygote deficiency (Table 3). The number of alleles per locus varied from 2 to 12, with an average of 4.8. MICROCHECKER discounted the presence of null alleles and, RMES analyses did not reveal biparental inbreeding in the regions (AL: ĝ_2_ = 0.081, s(ĝ_2_) = 0.235, p> 0.05 and; AP: ĝ_2_ = -0.0008, s(ĝ_2_) = 0, p> 0.05). Thus the Wahlund effect, the presence of two or more genetically differentiated populations in the sample arises as a possible cause, but the missing data may also have been responsible. Moreover, Fildes Bay has an absence of private alleles (S1 Table), while the mayor number is three and is shared by Rothera Base (West Antarctica) and Adélie Land (East Antarctica).

**Table 3:** Characterization of the genetic diversity of *Sterechinus neumayeri* for West and East Antarctic regions.

Regions	Loci	*N*	*A*	*H*_*o*_	*H*_*e*_	*F*_*IS*_
	F32St	55	3	0.073	0.071	-0.021
**West**	P1St	58	4	0.121	0.207	***0.425
**Antarctica**	P2St	51	12	0.471	0.578	**0.196
	N4St	47	4	0.617	0.697	*0.125
	V6St	57	6	0.561	0.457	-0.219
	F32St	12	3	0.250	0.226	-0.065
**East**	P1St	12	5	0.417	0.608	**0.353
**Antarctica**	P2St	11	5	0.091	0.508	***0.836
	N4St	11	4	0.818	0.727	-0.078
	V6St	10	2	0.200	0.180	-0.059

N: Number of individuals tested; A: Number of alleles; Ho: Observed heterozygosity; He: Expected heterozygosity; FIS: Weir and Cockerham's (1984) estimation of F_IS_

* = P < 0.05

** = P < 0.01

*** = P < 0.001.

#### Genetic structure analyses

Permutation tests did not detect any significant genetic structure between regions for the microsatellite data set, irrespective of whether individuals with missing data were included or not. In the single locus analyses, three loci presented significant genetic structure between regions, but the analyses without missing data only detected structure for a locus (Table 4). The Bayesian cluster analysis with STRUCTURE [102] detected a single genetic cluster (K = 1), for both runs, reinforcing the pattern of a very low or even lack of population structure between East and West Antarctic (S1 Fig).

**Table 4:** Permutation test between regions by locus for two data sets.

	A			B		
Loci	N	Fst	p	N	Fst	P
F32St	73	0.0385	**0.03**	39	0.1862	**0.02**
P1St	73	0.1510	**0.04**	39	0.0384	0.89
P2St	73	0.0130	0.62	39	0.0088	0.26
N4St	73	0.0233	0.89	39	0.0083	0.3
V6St	73	0.0634	**0.02**	39	0.0164	0.19
						
Total		0.0273	0.05		0.0142	0.19
(Multilocus)						

A) With missing data, B) Without missing data. N: Number of individuals tested; Fst: Fixation index; p: Significance value.

## Discussion

As previously proposed by Díaz et al. [31] for *S*. *neumayeri*, the genetic structure recorded in the species supports the presence of a single unit around the Antarctic continent. The extensive pattern of genetic homogeneity recorded in the mitochondrial gene (Geneland, cluster k = 1) was consistent with pattern observed using microsatellites. Similar patterns of genetic homogeneity over a broad geographical range have been reported in other groups of Antarctic invertebrates including *Parbolasia corrugatus* [34], *Chorismus antarcticus* [104] and *Nacella concinna* [32,33,49]. Moreover, *Parbolasia corrugatus* and *Chorismus antarcticus* shared haplotypes among the Antarctic Peninsula, Ross Sea and Weddell Sea [34,104]. The lack of genetic structure over large geographic distance can be explained by the presence of particular life-history traits including a long-lived larval stage with high dispersive potential [105]. Haye et al. [106] recognized that dispersal potential in terms of the pelagic larval duration (PLD) is an important determinant of the genetic structure of benthic marine species, which has been corroborated for some marine Antarctic species such as *Chorismus antarcticus*, *Nacella concinna* and *Nematocarcinus lanceopes* [33,104,107]. The dispersal ability of the pelagic stage of *S*. *neumayeri* lies in the prolonged free-living larval period recorded in the species, which allows transport over large distances [105]. Gene flow seems to limit the impact of genetic differentiation due to selection and/or genetic drift among distant areas. The extremely slow metabolism of *S*. *neumayeri* larvae described by Marsh et al. [67], could also contribute to extend its stay in the plankton (about a year, Manahan A. personal communication). Oceanographic currents might also play an important role in facilitating the genetic homogeneity through the transport of pelagic stages of Antarctic broadcasters like *S*. *neumayeri* [105]. Díaz et al. [31] proposed a model which includes two large routes of dispersion in the Southern Ocean, the Antarctic and Subantarctic two-ring model, that may maintain a degree of connectivity sufficient to prevent genetic and/or phylogeographic differentiation among populations: (1) the Antarctic Circumpolar Current (ACC—historically referred to as the West Wind Drift; [108]), which would connect the Sub-Antarctic zones, and (2) the Antarctic Costal Current (ACoC—East Wind Drift; [109]) which surrounds the Antarctic. According to our results, the high connectivity of *S*. *neumayeri* may have been facilitated principally by the current near-shore areas ACoC.

The remarkable characteristics of the extant shallow benthic fauna of the Southern Ocean fauna are its diversity and long-term persistence despite to the Quaternary glacial cycles [17,27,43,110]. The grounded ice sheets that extended over much of the Antarctic continental shelf during the glacial periods destroyed the available shelf habitat and forced the marine benthic fauna to move, adapt or go extinct [6,15,47,110,111]. The low levels of nucleotide polymorphism detected for *S*. *neumayeri* suggests that this species was strongly impacted by the recent Antarctic Quaternary climate history. Similar genetic diversity patterns have been reported in other Antarctic echinoderms including the irregular sea urchin *Abatus agassizii* [112], the brittle star *Ophionotus victoriae* [113], the sea star *Odontaster validus* [114] and the crinoid *Promachocrinus kerguelensis* [53], as well as in other marine invertebrates including the nemertean *Parbolasia corrugatus* [33], the malacostracan *Chorismus antarcticus* [103], amphipods of the genus *Eusirus* [115], the Antarctic limpet *Nacella concinna* [31], the sea spider *Nymphon australe* [29] and the benthic octopod *Pareledone turqueti* [116].

The possibility that a species with shallow distribution such as *S*. *neumayeri* may have survived in the deeper areas during the LGM is difficult to demonstrate or to test. However, the presence of the congeneric *S*. *antarcticus* on the outer Antarctic continental shelf (below 300 meters depth), and the restricted area in which both species do overlap [117] may suggest the existence of some competitive exclusion between them. The presence of *S*. *antarcticus* in deeper water may have impeded a habitat switch of *S*. *neumayeri* to less ice-impacted deeper areas during the LGM. Consequently, and together with the pattern of genetic diversity and structure detected in this study, the most plausible hypothesis explaining the resiliency of *S*. *neumayeri* in the Antarctic would be the *in situ* shelf refugia. Evidence for survival *in situ* during the glacial periods are common for the Northern Hemisphere, where some small ice-free refugia have been identified through the study of genetic diversity and structure. Maggs et al. [28] distinguished the genetic signature of recolonized areas from that of refugia by the presence of private alleles (i.e., alleles only occurring in one population resulting from isolation). In the Southern Hemisphere, some marine invertebrates would also have survived in one or several refugia on the Antarctic shelf; their location and number has been detected through their haplotype network pattern (for more details see [17]). The ‘star-like’ genealogy of *S*. *neumayeri* characterized by a single dominant haplotype around Antarctica together with numerous rare haplotypes differing by one or two substitutions is congruent with the pattern of genetic diversity proposed by Allcock & Strugnell [17] for species with a dispersal stage that survived the LGM in a single continental shelf refugium. This model considers rapid extinction of most populations, generating a considerable decrease in haplotype diversity of the species (eliminated polymorphism, [118]), while a single allele becomes fixed by genetic drift. Once the glacial maximum has passed and the ice sheets retreat, individuals with this allele becomes rapidly distributed across Antarctica. This pattern of a bottleneck event followed by rapid population expansion [119] has also been observed in the nemertean *Parbolasia corrugatus* [34] and the crustacean *Chorismus antarcticus* [104] that would have both persisted in a unique shelf refugium, while the Antarctic limpet *Nacella concinna* survived in South Georgia Island [33].

Ice-free regions existed in a range of temporal and spatial scales and are potential refuges during glacial cycles. Evidence for the diachrony of ice-sheet extensions around Antarctica resulting in the incomplete coverage of shelf areas by grounded ice [120] have been proposed to explain the persistence of bryozoans in the Weddell Sea [121,122]. On the other hand, Thatje et al. [47] suggested that singular areas of open water that persist in a glacial period, known as polynyas, could extend the period of primary production, thus allowing Antarctic shelf populations to persist during the LGM. These polynyas on the continental shelf edge have been described in the Weddell and Ross Seas [47,122]. The high haplotype diversity detected in the Weddell Sea in *S*. *neumayeri* is congruent with this hypothesis, but in the Antarctic region, Adelie Land (East Antarctica) displayed the highest nucleotide diversity (0.00115) and suggests this area as a potential refuge for the species. This area has also been proposed as glacial refugium for the crinoid with a planktonic stage *Promachocrinus kerguelensis* [54], amphipods of the genus *Eusirus* [115], the sea spider *Nymphon australe* [30] and the benthic octopod *Pareledone turqueti* [116]. Parts of East Antarctica may have been permanently free of grounded ice during the LGM [120]. The presence of such refugia is also suspected off George V Land [123] due to the peculiarity of the shallow water fauna in this locations compared to those of other areas. Also, in Windmill Islands (a group of rocky islands in East Antarctica) the ice sheet was much thinner and some areas may have remained unglaciated during at least the Holocene period [124].

During the Early-Middle Holocene [125], the deglaciation scenario in Antarctica was not synchronous, but most dates from the inner shelf areas and fjords and bays constrain deglaciation to as late as 9–6 ka BP [126]. For example, in King George Island (west peninsula), the estimated age for initial deglaciation is 9–8 ka BP [126,127,128], while in James Ross Island (East Antarctic Peninsula), the onset of deglaciation is estimated before ca. 7.4 ka BP [126,128,129]. For East Antarctica, the deglaciation was already ongoing in the early Holocene, while in the Windmill Islands, Bunger Hills, and Lambert Glacier area the ice was already at its present position by 8–6 ka BP [129]. This deglaciation process along inner shelf areas around Antarctica in the Early-Middle Holocene provides a realistic frame for a rapid post LGM population expansion of *S*. *neumayeri*, mainly through the main oceanographic currents that allow long distance dispersal of pelagic larvae around the Southern Ocean [31,37,130,131]. The estimated date of population expansion applying the 10-fold correction infers a period in the middle Holocene, between ~4.8–6.6 ky, while the most recent common ancestor for the lineage occurred ~ 6.0–8.2 ky (considering mutation rates of 5.1% and 7.2%, respectively). These dates fit well with accepted hypotheses of benthic invertebrates survival in refugia and population expansions associated with deglaciation events after the LGM [32,36,132] and with other Antarctic invertebrates such as *Nacella concinna* [33], *Nematocarcinus lanceopes* [104], *Nymphon austral* [133] and *Limacina antarctica* [134].

## Conclusions

Climatic and oceanographic processes together with life history traits are major factors explaining the evolutionary history of *S*. *neumayeri* in the Southern Ocean and some unique characteristics like the endemism and extensive distribution along the Antarctic shelf. Our results suggest a circum-Antarctic connectivity strong enough to impede any divergence processes around the continent. Under a postglacial scenario of recent demographic expansion, our results also suggested that the extension of ice sheets during the LGM may have drastically reduced the habitat and population sizes of *S*. *neumayeri*. Because *S*. *neumayeri* is endemic to the upper Antarctic continental shelf, the star-like topology with a central dominant haplotype, low genetic diversity and recent demographic expansion signal supports the hypothesis of a single *in-situ* refugium. We propose a scenario of rapid postglacial recolonization of shallow Antarctic areas from one area less ice-impacted where it survived through the LGM, probably in the eastern part of the continent.

## Supporting information

S1 Table Number of private alleles by locus for locality. (DOCX)

S1 Fig Bayesian analysis results of *Sterechinus neumayeri* from structure. A) Boxplot for the assignment of individual genotypes for K = 1 to K = 5; B) Graph that shows the variation of the mean Ln probability of data.
(TIF)
