# Supplementary material for: Genetic structure and demographic inference of the regular sea urchin Sterechinus neumayeri (Meissner, 1900) in the Southern Ocean: The role of the last glaciation
Source: PLoS One. 2018 Jun 6;13(6):e0197611. doi: 10.1371/journal.pone.0197611 (PMC5991379; doi:10.1371/journal.pone.0197611)
Supplement: S1 Table — (DOCX) [file pone.0197611.s001.docx]

|  | **Loci** | | | | |  |
| --- | --- | --- | --- | --- | --- | --- |
| **Locality** | **F32St** | **P1St** | **P2St** | **N4St** | **V6St** | **TOTAL** |
| **R_1_** Antarctic Peninsula |  |  |  |  |  |  |
| J. Ross Island | - | - | - | - | 1 | 1 |
| Covadonga Bay | - | - | 1 | - | 1 | 2 |
| Paradise Bay | 1 | - | 1 | - | - | 2 |
| Rothera Base | - | - | 3 | - | - | 3 |
| Fildes Bay | - | - | - | - | - | 0 |
| **R**_2_ Adélie Land | 1 | 1 | 1 | - | - | 3 |
