# Supplementary figures and images for: Genetic structure and demographic inference of the regular sea urchin Sterechinus neumayeri (Meissner, 1900) in the Southern Ocean: The role of the last glaciation
Source: PLoS One. 2018 Jun 6;13(6):e0197611. doi: 10.1371/journal.pone.0197611 (PMC5991379; doi:10.1371/journal.pone.0197611)

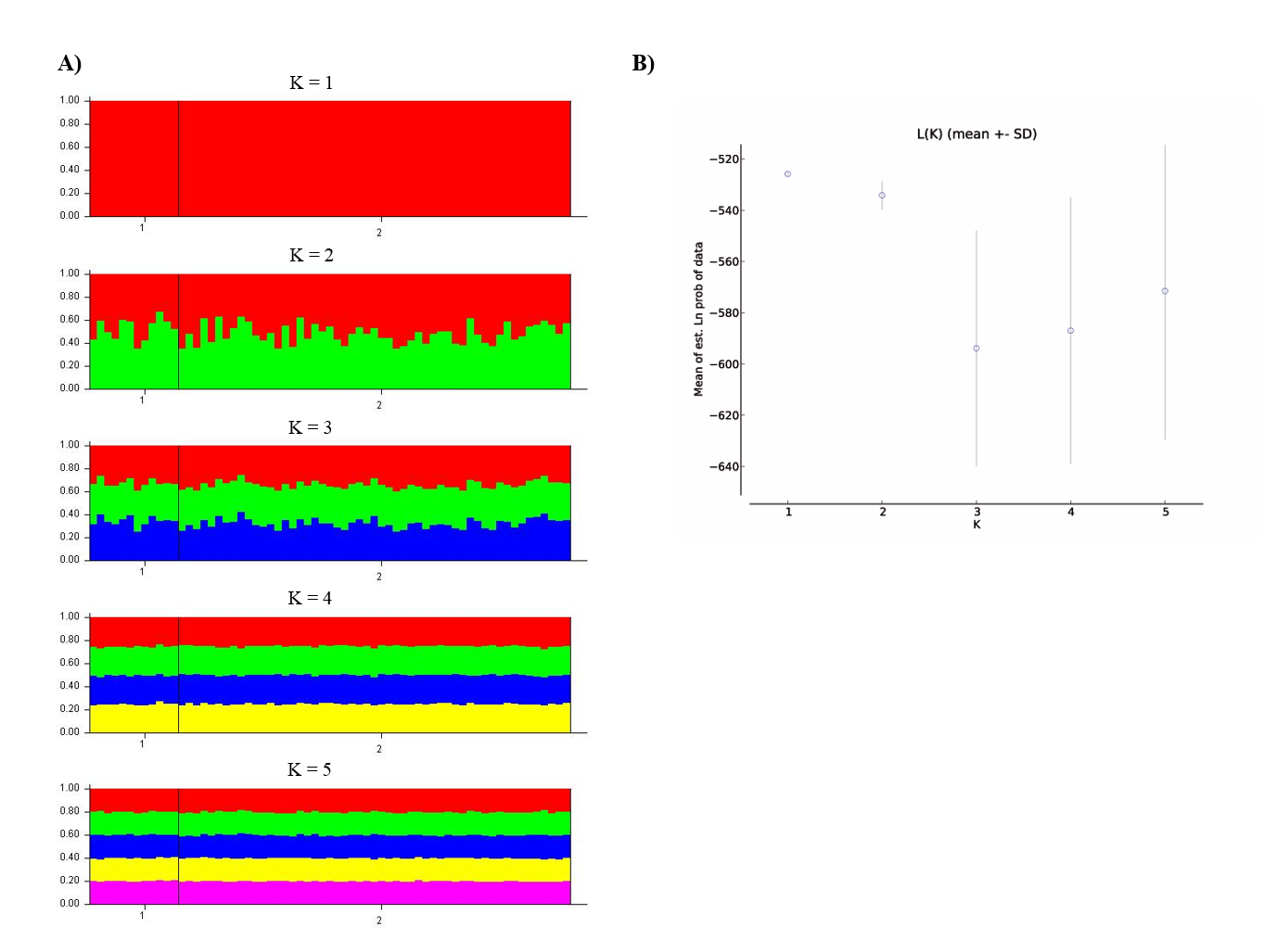

Supplement: S1 Fig — A) Boxplot for the assignment of individual genotypes for K = 1 to K = 5; B) Graph that shows the variation of the mean Ln probability of data. (TIF) [file pone.0197611.s002.tif]
